# Serological screening for specific antibodies against TORCH pathogens in reproductive-aged women in Zhangzhou, China

**DOI:** 10.3389/fpubh.2025.1674430

**Published:** 2025-12-18

**Authors:** Pengfei Huang, Weide Chen, Guowei Wang, Yuanjun Zeng, Yueli Guo

**Affiliations:** 1 Collaborative Innovation Center for Translation Medical Testing and Application Technology, Department of Medical Technology, Zhangzhou Health Vocational College, Zhangzhou, Fujian, China; 2 Department of Laboratory Medicine, Zhangzhou Affiliated Hospital of Fujian Medical University, Zhangzhou, Fujian, China

**Keywords:** TORCH pathogens, seropositivity, reproductive-aged women, Herpes simplex virus, China

## Abstract

**Introduction:**

TORCH is a group of pathogens including Herpes simplex virus (HSV), Cytomegalovirus (CMV), Rubella virus (RV) and Toxoplasma gondii (TOX). Serological screening for the specific antibodies against TORCH pathogens is crucial for preventing fetal malformation and miscarriage. In this study, we analyzed the characteristics of TORCH IgM and IgG seropositivity in reproductive-aged women in Zhangzhou, China.

**Methods:**

A total of 1,417 reproductive-aged women prior to conception attended prenatal diagnosis outpatient clinic in Zhangzhou and received the prenatal TORCH serological screening were enrolled in this study. The IgM and IgG antibodies against TORCH pathogens were detected using chemiluminescence immunoassay.

**Results:**

The positive rates of IgM for TOX, RV, CMV and HSV-1/2 were 0.64, 2.33, 1.34 and 11.22%. The IgG seropositivity for TOX, RV, CMV, HSV-1 and HSV-2 were 3.81, 72.83, 97.46, 82.64 and 7.27%. The positive rate of HSV-1/2-IgM and TOX-IgG in 2024 were lower than that in 2023. In addition, the positive rates of RV-IgG and HSV-1-IgG were significantly higher in the women aged 30–34 years old and ≥35 years old. The CMV-IgM and HSV-2-IgG positive rates were higher in women ≥35 years old. Regarding seasonal distribution, the positive rate of HSV-1/2-IgM was significantly higher in spring (March to May) than that in winter (December to February). The seropositivity of all TORCH-IgG showed no significant differences across four seasons.

**Conclusion:**

Our research highlights the age and seasonal distribution of TORCH seroprevalence in reproductive-aged women in Zhangzhou. These findings underscore that continuous serological surveillance is important for the prevention of congenital infections caused by TORCH pathogens.

## 1 Introduction

The representative TORCH pathogens include Herpes simplex virus (HSV), Cytomegalovirus (CMV), Rubella virus (RV) and Toxoplasma gondii (TOX). Acute TORCH infections during pregnancy are associated with increasing risk of miscarriage and congenital abnormalities (1). TORCH pathogens may transmit vertically through placenta and pose great threat to fetus (2). Previous reports revealed that 2–3% of all neonatal infections and congenital abnormalities were caused by TORCH vertical infection (3). However, most pregnant women infected with TORCH pathogens are asymptomatic or in a subclinical state. Thus, the TORCH prenatal screening in reproductive-aged women is vital for reducing the incidence of miscarriage and fetal malformation (4).

The serological tests for detecting TORCH IgM and IgG are crucial for diagnosis of acute or past TORCH infection. The TORCH-IgG screening also contributes to evaluate the vaccination and immunity status. Chinese Medical Association recommended that the TORCH screening should be carried out within 3 months before pregnancy since 2011; and continuous TORCH serological surveillance is necessary for controlling TORCH infection (4).

There are a few reports about the TORCH seroprevalence in different areas of China, including Beijing city (5), Henan province and northwest China (6, 7). These researches have underscored the TORCH infection burden on reproductive-aged women in China. However, the TORCH seroprevalence showed significant regional disparities across different areas, which have been identified to be caused by geographical location, population distribution and vaccination coverage (8–10). Zhangzhou city is located in the southeastern China. There is still lack of analysis on the TORCH IgM and IgG seroprevalence data in Zhangzhou city and its adjacent areas, which further hampers implementation of preventative measures.

In this study, we carried out serological screening for the TORCH specific IgM and IgG antibodies in reproductive-aged women in Zhangzhou, China. Particularly, we focused on the distribution of TORCH IgM and IgG seropositivity in different age groups and seasons. This research aims to provide valuable insights into the formulation of TORCH preventive measures.

## 2 Materials and methods

### 2.1 Ethics statement

The study was approved by the Ethics Committee of Zhangzhou Affiliated Hospital of Fujian Medical University. All experiments were conducted according to the local legislation and institutional requirements. Considering the retrospective nature of this research, Institutional Review Board waived the requirement of informed consent. This study was conducted in accordance with the basic principles of the Declaration of Helsinki.

### 2.2 Study subjects

The retrospective study was conducted from January 2023 to December 2024 at the Zhangzhou Affiliated Hospital of Fujian Medical University.

#### 2.2.1 Inclusion criteria

A total of 1,417 reproductive-aged women prior to conception attended prenatal diagnosis outpatient clinic in this hospital and received prenatal TORCH serological screening were enrolled as study subjects. These hospital-based samples represented the general population who underwent the prenatal screening in hospital.

#### 2.2.2 Exclusion criteria

The women who were previously diagnosed as infertility were excluded. The repeat samples from same individuals and cases with incomplete data were excluded from the study.

The age range of study subjects was 16–50 years. The subjects were divided into 4 age groups, including ≤24 years old, 25–29 years old, 30–34 years old, and ≥35 years old; and divided into spring (March–May), summer (June–August), autumn (September–November), winter (December–February). Sample collection was carried out by trained personnel in accordance with standard operating procedures and stored at −70 °C prior to analysis.

### 2.3 Reagents and methods

The peripheral venous blood from reproductive-aged women were collected and centrifuged to collect serum. The samples were analyzed according to the manufacturer’s instructions using indirect chemiluminescence immunoassay (CLIA) technology, Liaison XL platform from DiaSorin.

Reagents used in this study: LIAISON® Rubella IgM reagent (310,730, DiaSorin S. P. A., Italy), LIAISON® HSV-1/2-IgM reagent (310,820, DiaSorin S. P. A., Italy), LIAISON® Toxo-IgM reagent (310,710, DiaSorin S. P. A., Italy), LIAISON® CMV-IgM reagent (310,755, DiaSorin S. P. A., Italy), LIAISON® Rubella-IgG reagent (310,720, DiaSorin S. P. A., Italy), LIAISON® HSV-2-IgG reagent (310,810, DiaSorin S. P. A., Italy), LIAISON® CMV-IgG reagent (310,740, DiaSorin S. P. A., Italy).

### 2.4 Quality control

LIASON control IgM/IgG for TOX, RV, CMV and HSV-1/2 reagents (055056, DiaSorin S. P. A., Italy) were applied for the quality control. The positive and negative quality control samples were loaded on the Liaison XL platform. And then the quality control samples were detected according to the manufacturer’s instructions. The quality control samples with different concentrations should be tested at least once every 24 h.

### 2.5 Judgment criteria

1. TORCH-IgM: The cut-off value of TOX-IgM, RV-IgM, CMV-IgM and HSV-1/2-IgM were 3.00 AU/mL, 10.00 AU/mL, 5.00 U/mL and 0.50 AU/mL, respectively.
2. TORCH-IgG: The cut-off value of TOX-IgG, RV-IgG, CMV-IgG and HSV-1-IgG and HSV-2-IgG were 3.00 AU/mL, 3.00 AU/mL, 1.00 AU/mL, 0.5 AU/mL and 0.5 AU/mL, respectively.

### 2.6 Statistical analysis

Statistical analyses were conducted by SPSS software version 23 (SPSS Inc. Chicago, IL, USA). For the inter-group comparisons across different age and seasons groups, we performed Chi-square test or Fisher’s exact test to determine the significance. And *p* < 0.05 was considered as statistically significance.

For pairwise comparisons between multiple groups, the test level was corrected by the Bonferroni method. The adjusted test level for pairwise comparisons between different age groups was *p* = 0.05/6 = 0.0083, and the adjusted test level for pairwise comparisons between different seasons was *p* = 0.05/6 = 0.0083.

## 3 Results

### 3.1 The overall positive rates of TORCH IgM and IgG

From January 2023 to December 2024, a total of 1,417 cases were enrolled. In addition, a total of 426 samples were collected in spring (March–May), 337 in summer (June–August), 309 in autumn (September–November) and 345 in winter (December–February). All cases were divided into 4 age groups, including (1) ≤24 years old: 240 cases; (2) 25–29 years old: 504 cases; (3) 30–34 years old: 428 cases; (4) ≥35 years old: 245 cases.

Among 1,417 samples, 220 samples (15.25, 95% CI: 13.64–17.41) were positive for at least one TORCH IgM antibodies. Ten samples were positive for two TORCH IgM antibodies (0.7, 95% CI: 0.265–1.134). As shown in Table 1, the IgM seropositivity for TOX, RV, CMV and HSV-1/2 were 0.64% (95% CI: 0.22–1.05), 2.33% (95% CI: 1.54–3.11), 1.34% (95% CI: 0.74–1.93) and 11.22% (95% CI: 9.58–12.86). The positive rate for HSV-1/2-IgM was the highest (11.22%, 159/1417) followed by RV-IgM (2.33%, 33/1417) and CMV-IgM (1.34%, 19/1417).

**Table 1:** Overall seropositivity rate of TORCH IgM and IgG in reproductive-aged women.

Pathogen	IgM			Pathogen	IgG		
	*N* ^a^	*n* ^b^	Positive rate% (95% CI)		*N* ^a^	*n* ^b^	Positive rate % (95% CI)
TOX	1,417	9	0.64 (0.22–1.05)	TOX	1,417	54	3.81 (2.81–4.80)
RV	1,417	33	2.33 (1.54–3.11)	RV	1,417	1,032	72.83 (70.51–75.15)
CMV	1,417	19	1.34 (0.74–1.93)	CMV	1,417	1,381	97.46 (96.64–98.28)
HSV-1/2^c^	1,417	159	11.22 (9.58–12.86)	HSV-1	1,417	1,171	82.64 (80.67–84.61)
				HSV-2	1,417	103	7.27 (5.92–8.62)

^a^Number of total cases. ^b^Number of positive cases. ^c^HSV-1/2 represents the total seropositivity rate of HSV-1-IgM and HSV-2-IgM.

The total IgG seropositivity rates for TOX, RV, CMV, HSV-1 and HSV-2 were 3.81% (95% CI: 2.81–4.80), 72.83% (95% CI: 70.51–75.15), 97.46% (95% CI: 96.64–98.28), 82.64% (95% CI: 80.67–84.61) and 7.27% (95% CI: 5.91–8.62). The positive rate for CMV-IgG was highest (97.46%, 1381/1417) followed by HSV-1-IgG (82.64%, 1171/1417) and RV-IgG (72.83%, 1032/1417).

**Figure 1:**
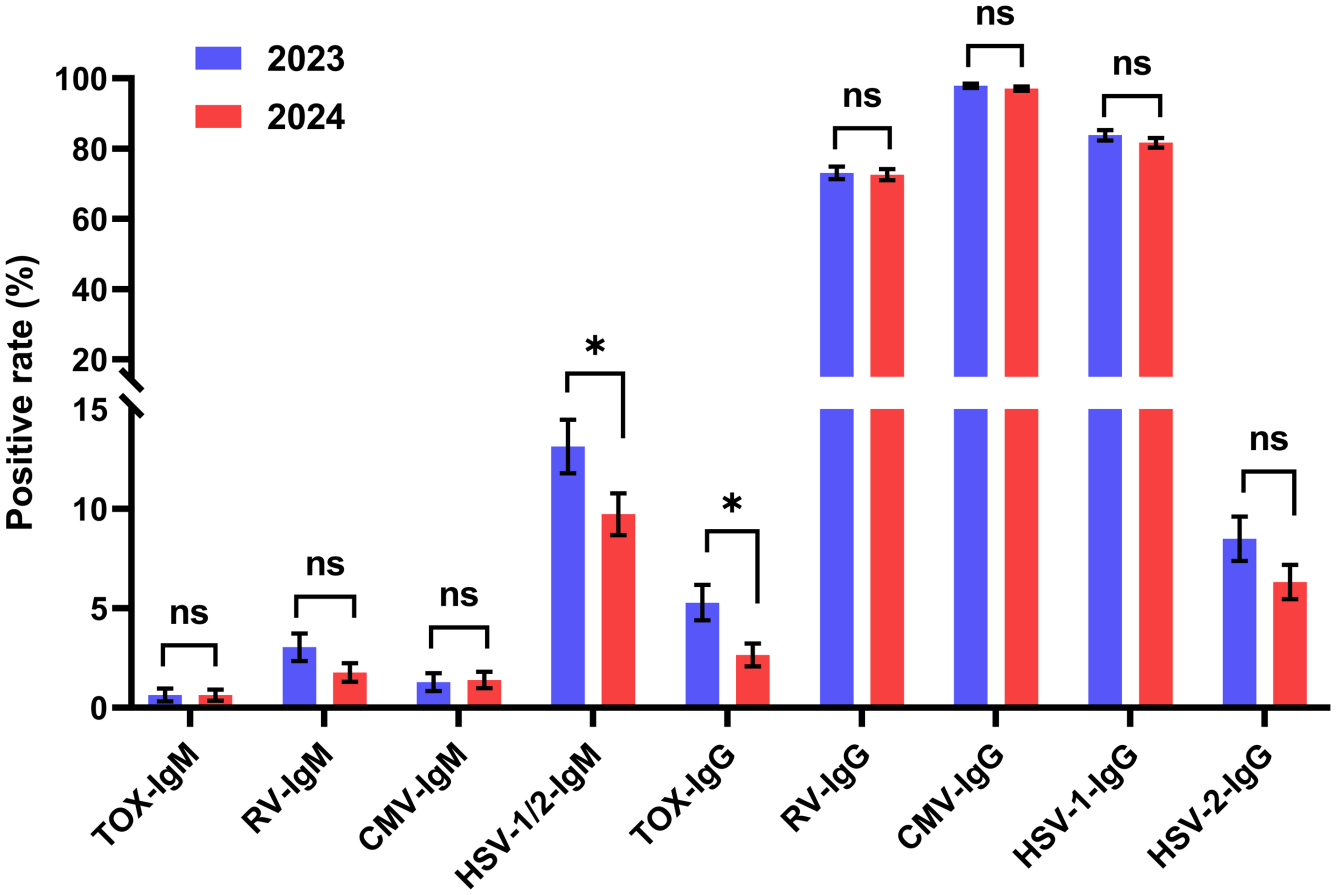
Distribution of TORCH-IgM/IgG seropositivity rates in 2023 and 2024. Chi-square tests or Fisher’s exact tests were performed to determine the significance. The error bars represented 95% confidence intervals of positive rate. *p < 0.05, **p < 0.01, ns, no significant. Blue: 2023, red: 2024.

During January 2023 to February 2024, 625 samples were collected in 2023 and 792 samples were collected in 2024. The positive rates of HSV-1/2-IgM and TOX-IgG in 2024 were significantly lower than that in 2023 (Figure 1). While no significant differences were observed in other TORCH IgM/IgG between 2023 and 2024.

### 3.2 Distribution of TORCH IgM and IgG positive rates across different age groups

**Figure 2:**
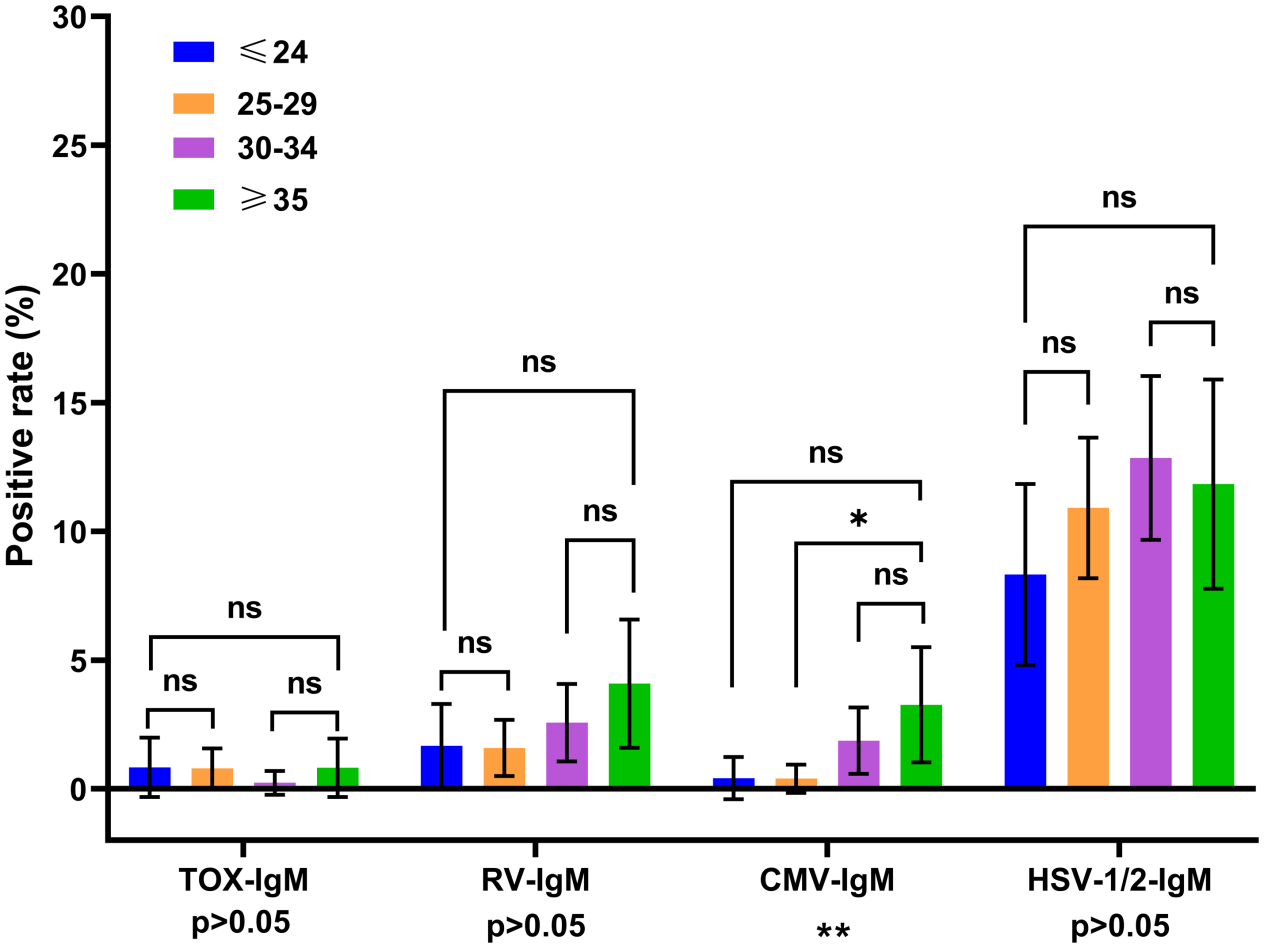
Distribution of TORCH-IgM seropositivity rates across different age groups. The error bars represented 95% confidence intervals of positive rate. The *p* value of inter-group comparisons across all different age groups was shown under the X axis; For inter-group comparisons: *p < 0.05, **p < 0.01, ns, no significant. The *p* value of the pairwise comparisons between different age groups were shown above the columns. Fisher’s exact tests or Chi-square were performed to determine the significance of pairwise comparison; and the *p* value was corrected by Bonferroni method. For pairwise comparisons: *p < 0.00833, **p < 0.00167, ns: no significant. Blue: ≤24 years, orange: 25–29 years, purple: 30–34 years, green: ≥35 years.

**Figure 3:**
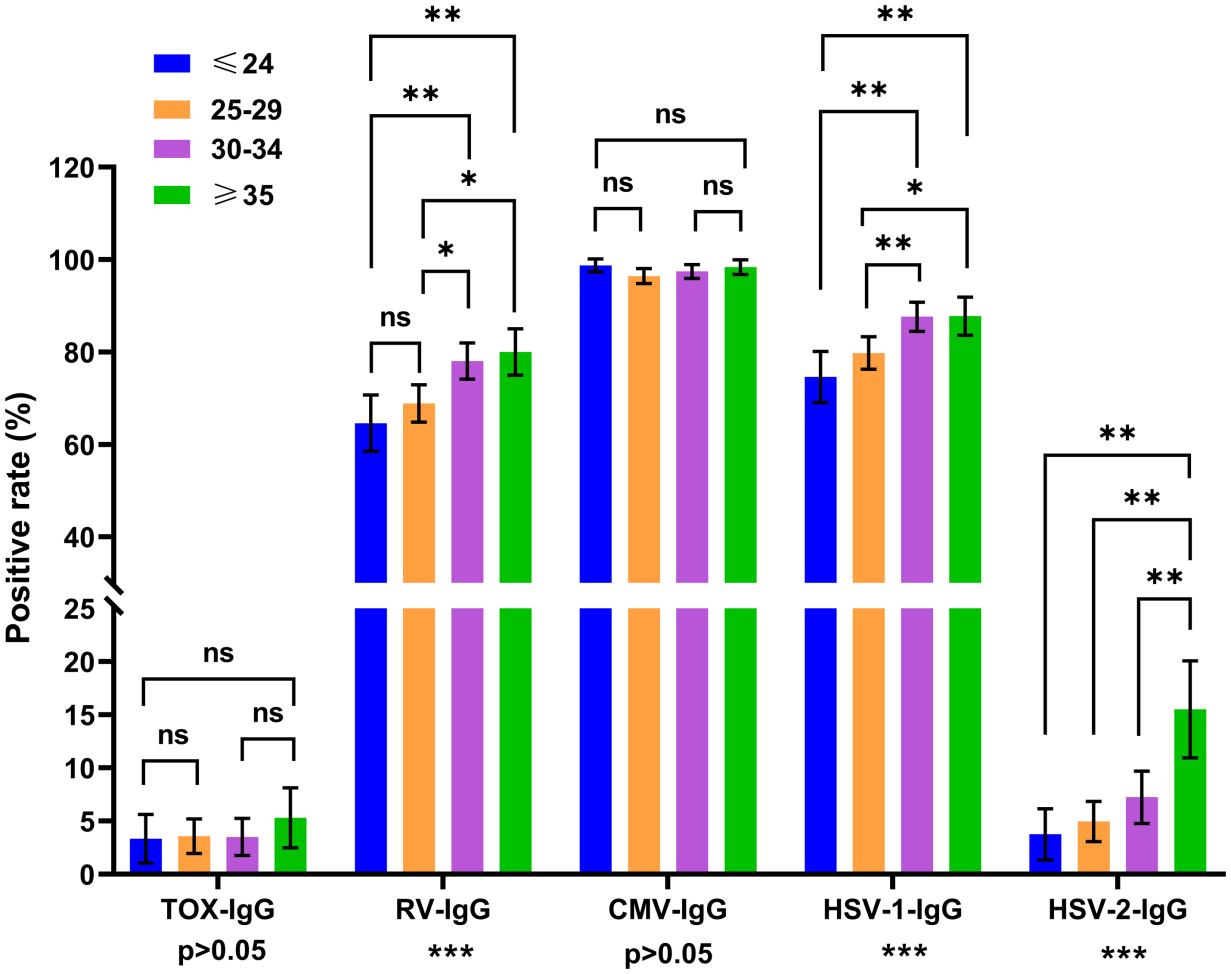
Distribution of TORCH-IgG seropositivity rates across different age groups. The error bars represented 95% confidence intervals of positive rate. The *p* value of inter-group comparisons across all different age groups was shown under the X axis. For inter-group comparisons: *p < 0.05, **p < 0.01, ***p < 0.001, ns, no significant. The p value of the pairwise comparisons between different age groups were shown above the columns. Fisher’s exact tests or Chi-square were performed to determine the significance of pairwise comparison; the *p* value was corrected by Bonferroni method. For pairwise comparisons: *p < 0.00833, **p < 0.00167, ns: no significant. Blue: ≤24 years, orange: 25–29 years, purple: 30–34 years, green: ≥35 years.

To analyze the age distributions of TORCH infections, all women of reproductive age were divided into 4 age groups. For the inter-group comparison across different age groups, the positive rate of HSV-IgM, RV-IgM and TOX-IgM did not show statistically significance across different age groups (*p* > 0.05). While the positive rate of CMV-IgM showed significant difference across different age groups (*p* < 0.01) (Figure 2). As shown in Figure 3, the positive rates of RV-IgG, HSV-1-IgG and HSV-2-IgG exhibited significant differences among different age groups (all *p* < 0.001).

For the pairwise comparison, the *p* value was corrected by Bonferroni methods (*p* < 0.00833 was considered as significant difference). The CMV-IgM seropositivity in women ≥35 years old was significantly higher than that of women aged 25–29 years old (*p* < 0.00833). The positive rate of RV-IgG was significantly higher in women aged 30–34 and ≥35 than those aged ≤24 (both *p* < 0.00167) and 25–29 (both *p* < 0.00833). While no significant difference was observed between the 30–34 years old and ≥35 years old women (*p* > 0.00833). The positive rates of HSV-1-IgG in the women aged 30–34 and ≥35 were higher than those aged ≤24 (both *p* < 0.00167), and 25–29 (*p* < 0.00833). The positive rate of HSV-2-IgG was higher in the women aged ≥35 than three other age groups (all *p* < 0.00167). The positive rate of TOX-IgG and CMV-IgG exhibited no significant differences across different age groups (Figure 3).

### 3.3 Distribution of TORCH IgM and IgG positive rates across different seasons

**Figure 4:**
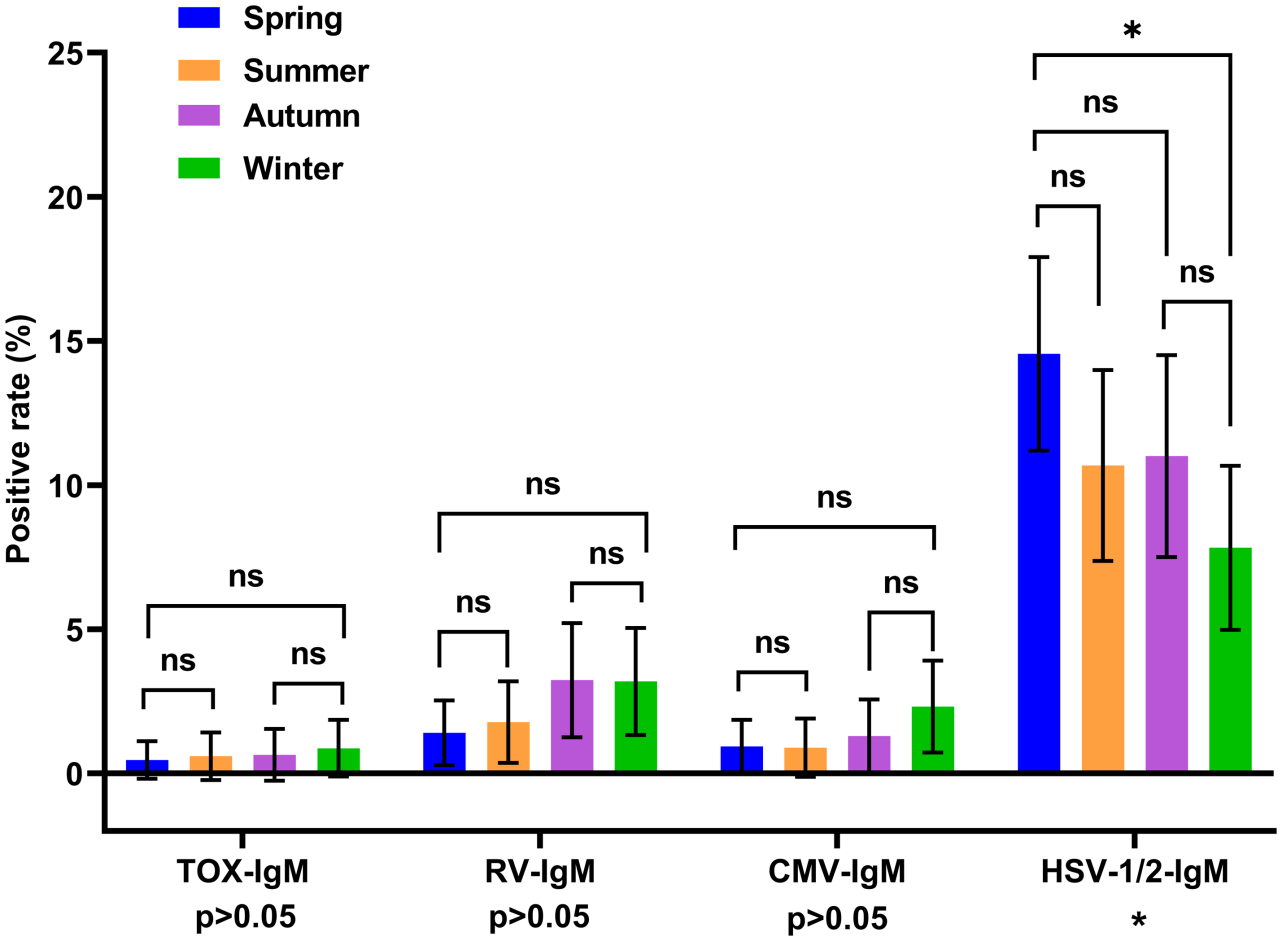
Distribution of TORCH-IgM seropositivity rates across different seasons. The error bars represented 95% confidence intervals of positive rate. The *p* value of inter-group comparisons across different seasons was shown under the X axis. For inter-group comparisons: *p < 0.05, **p < 0.01, ns, no significant. The *p* value of pairwise comparisons between different seasons were shown above the columns. Fisher’s exact tests or Chi-square were performed to determine the significance of pairwise comparison; and the *p* value was corrected by Bonferroni method. For pairwise comparisons: *p < 0.00833, **p < 0.00167, ns, no significance. Blue: spring, orange: summer, purple: autumn, green: winter. Spring: March to May, Summer: June to August, Autumn: September to November, Winter: December to February.

**Figure 5:**
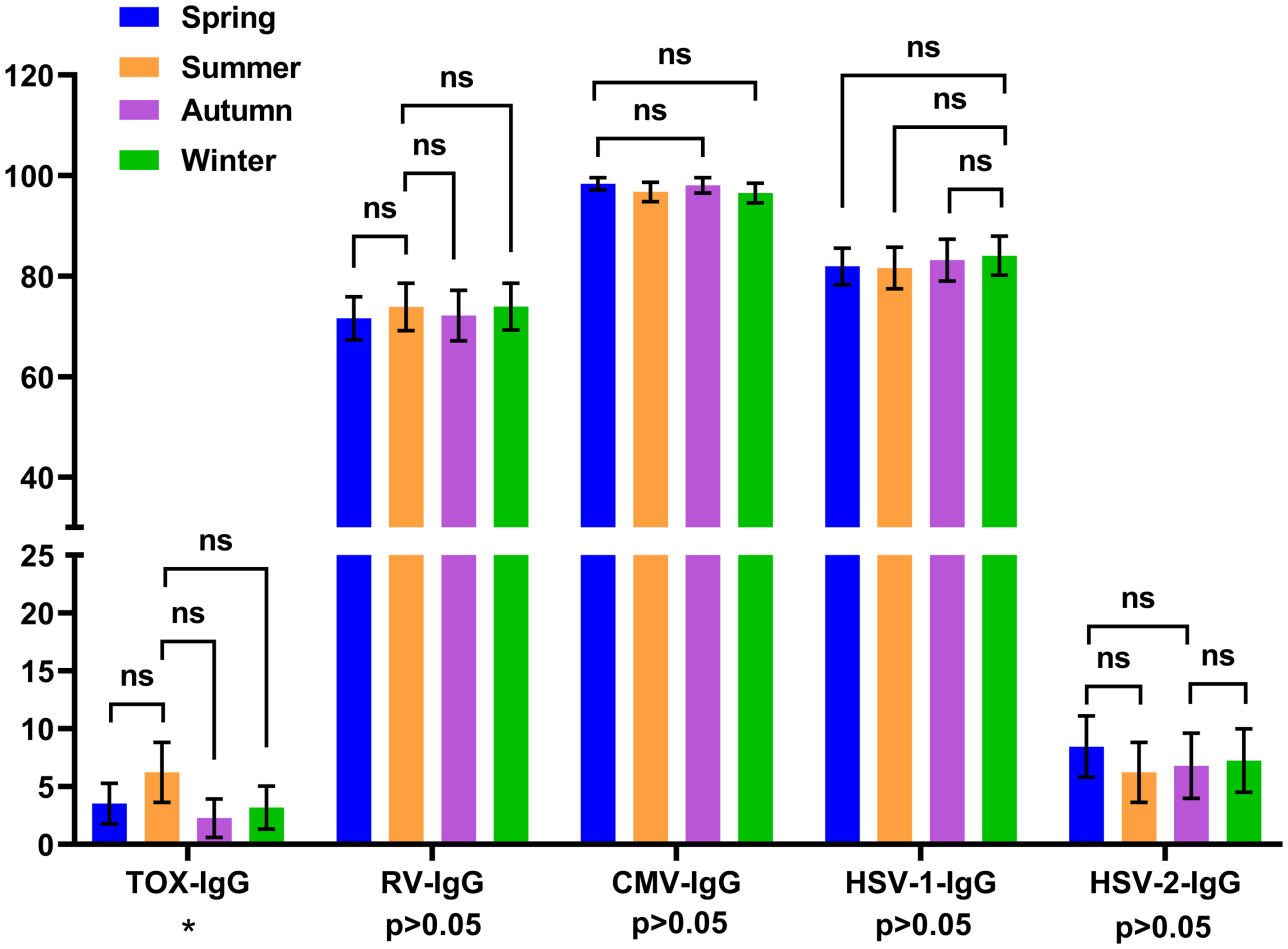
Distribution of TORCH-IgG seropositivity rates across different seasons. The error bars represented 95% confidence intervals of positive rate. The *p* value of inter-group comparisons across different seasons was shown under the X axis. For inter-group comparisons: *p < 0.05, **p < 0.01, ns, no significant. The *p* value of pairwise comparisons between different seasons were shown above the columns. Fisher’s exact tests or Chi-square were performed to determine the significance of pairwise comparison; and the *p* value was corrected by Bonferroni method. For pairwise comparisons: *p < 0.00833, **p < 0.00167, ns, no significant. Blue: spring, orange: summer, purple: autumn, green: winter. Spring: March to May, Summer: June to August, Autumn: September to November, Winter: December to February.

To validate the seasonal distribution of TORCH seropositivity, we performed pairwise comparisons across different seasons. And *p* values were corrected by Bonferroni method. The positive rate of HSV-1/2-IgM was significantly higher in spring (March–May) than that in winter (December–February) (*p* < 0.00833). While the positive rates of RV-IgM, CMV-IgM and TOX-IgM showed no significant differences across four seasons (Figure 4). The inter-group comparison of TOX-IgG positive rate across different seasons showed significant difference (*p* = 0.0489 < 0.05). However, there was no significant difference in the pairwise comparisons of TOX-IgG seropositivity between multiple seasons after the correction of *p* value (*p* > 0.00833). The positive rate for the RV-IgG, CMV-IgG, HSV-1-IgG and HSV-2-IgG did not differ across four seasons (Figure 5).

## 4 Discussion

TORCH infection is the crucial cause of miscarriage and fetal malformation, which poses a serious public health threat to pregnant women and fetus. Thus, the TORCH infection should be avoided before and during pregnancy (10, 11). Early seropositivity screening of TORCH IgM and IgG antibodies is crucial for the prevention of TORCH pathogens (12). In Zhangzhou, China, the lack of TORCH serological screening data prompted us to execute a comprehensive analysis for the local seropositivity of TORCH pathogens. In this study, we conducted TORCH-IgM/IgG serological screening among reproductive-aged women before pregnancy in Zhangzhou, then analyzed the age or seasonal distributions of TORCH-IgM/IgG seropositivity.

In our study, the IgM seropositivity of TOX, RV, CMV and HSV-1/2 were 0.64, 2.33, 1.34 and 11.22%, respectively. And the seropositivity of HSV-IgM was the highest (Table 1). Chen et al. used enzyme linked immunosorbent assay (ELISA) to screen the TORCH IgM and IgG in 10,669 women before pregnancy in Beijing (5). They demonstrated positivity rates for TOX-IgM (0.67%), RV-IgM (2.55%), CMV-IgM (1.24%) and HSV-1/2 (8.24%). These data indicated that the positive rate of HSV-IgM in Beijing was significantly lower than that in our research. Ren and et al. carried out serological screening in 17,807 infertile women in northwest China (6), with the positive rate for TOX-IgM (0.46%), RV-IgM (0.77%), CMV-IgM (0.68%) and HSV-1/2-IgM (1.82%). However, the positive rate of RV-IgM (2.33%) and HSV-IgM (11.22%) in our research were far higher than that in northwest China. These results indicated that TORCH IgM positive rate in different regions exhibited disparate results, which might be related to different geographical location, population distribution and personal lifestyles (11, 13). The higher RV-IgM positive rate in Zhangzhou might resulted from the low vaccination coverage. In addition, the higher positive rate of HSV-IgM might be caused by the relative high infection rate in Zhangzhou or the false-positive rates of our tests which need further validation.

The IgG positive rate of TOX, CMV, HSV-1 and HSV-2 in our research were similar to the previous researches (5). However, the RV-IgG positive rate (72.83%) in Zhangzhou was lower than that in Beijing (89.74%) and Northwest China (84.93%). The discrepancies of RV-IgG across different regions of China may be related to the different vaccination coverage. Vaccination of RV is crucial for preventing congenital RV infection. However, vaccination coverage of reproductive-aged women in Zhangzhou is still not optimistic, which lead to a low positive rate of RV-IgG (14, 15). Therefore, we recommended to strengthen the RV antibodies screening and optimize the vaccine program for reproductive-aged women. In addition, The HSV-1-IgG and CMV-IgG were both at high levels, indicating that the women in Zhangzhou were generally susceptible to HSV and CMV and exhibited desirable immunity level.

We also analyzed the distribution of TORCH-IgM/IgG seroprevalence in 2023 and 2024. The positive rates of HSV-1/2-IgM and TOX-IgG in 2024 were significantly lower than that of 2023 (Figure 1). The possible reason could be that people’s awareness for TORCH prevention has gradually increased. The reproductive-aged women have paid more attention to strengthen the prenatal TORCH screening and maintain good lifestyle habits.

Our findings demonstrated that the seropositivity of CMV-IgM, RV-IgG, HSV-1-IgG and HSV-2-IgG showed significantly differences across all age groups (Figures 2, 3). The seroprevalence of CMV-IgM was significantly higher in the women aged ≥35 groups. The possible reason could be that the immunity against CMV infection gradually declined with age and resulted in the reactivation of CMV infection in the older women. The RV-IgG positive rate was statistically lower in ≤24 years old and 25–29 years old groups. The possible reason could be that the RV vaccination coverage in this population is relatively low. Therefore, an optimized RV vaccination program should be implemented to increase the vaccination rate of young women in Zhangzhou. In addition, serological tests in the pre-pregnancy phase are necessary for the evaluation of immunity status against RV infection.

Our study revealed that the positive rate of HSV-1-IgG was significantly higher in women aged 30–34 and ≥35 years old. In addition, the HSV-2-IgG seropositivity was significantly higher in women aged ≥35 years old. The HSV-1 and HSV-2 IgG positive rate showed an elevated tendency with age, which may be caused by the distinct exposure time of HSV-1 and HSV-2 in different age groups. The age distributions of HSV-1-IgG and HSV-2-IgG in this study were similar to previous research in Northwest China (6). These results indicated that infection rate of HSV-1/HSV-2 were at a high level in multiple areas of China, especially in older adults groups. Thus, the serological screening of HSV-1/ HSV-2 seroprevalence is crucial for prevention of primary HSV infections and severe neuro logical damage caused by HSV.

We also analyzed the seasonal distribution of TORCH IgM and IgG seropositivity rate. Our results revealed that HSV-IgM positive rate in spring was significantly higher than that in winter. While CMV-IgM, RV-IgM, TOX-IgM, and all five TORCH IgG positive rates showed no significantly difference across four seasons (Figures 4, 5). Another research in Beijing revealed that the HSV-IgM was significantly higher in autumn and winter, which was different from our results. The difference might be related to the distinct population distribution, lifestyle habits and distinct climate between these two regions (16–18). These data above revealed that seropositivity of HSV-IgM showed distinct seasonal distributions across different regions. Thus, we recommended to enhance continuous prenatal TORCH surveillance and propose seasonal-tailored preventive interventions to reduce the TORCH infection burdens. The positive rate of TOX-IgG showed statistically difference across different seasons. However, there was no significant difference in the pairwise comparisons after Bonferroni correction of *p* value.

There are several limitations in this study. Firstly, the subjects enrolled in this research all came from a single geographic region, the results obtained in this research might not be applicable for other geographical regions with distinct population distribution and lifestyle habits. Multi-center research should be further carried out. Secondly, this research lacked correlation analysis between positive rates and clinical outcomes, which could provide further cognition of TORCH infections to women. Third, there was hospital-based sample bias in our research. The positive rates of this research were obtained from hospital-based sample which may be unable to represent the positive rate of healthy people in the community. Fourth, our study did not take account of the possible impact of confounding factors such as pregnancy status and parity on the TORCH seropositivity.

## 5 Conclusion

This research provided baseline serological surveillance data for the seropositivity of TORCH specific IgM and IgG among reproductive-aged women in Zhangzhou. Based on this research, it could be concluded that the seroprevalence of CMV-IgG and HSV-1-IgG were at high levels, indicating desirable population immunity level against these pathogens in Zhangzhou. The positive rate of RV-IgG was relatively low in young women, which suggested that the vaccination strategy optimization was necessary.

In addition, the HSV-1/2-IgM seropositivity was comparatively high in Zhangzhou especially in the spring, indicating that the risk of HSV infection among reproductive-aged women in Zhangzhou were still high. Our research highlighted that continuous serological surveillance, vaccination program optimization and seasonal-tailored intervention were crucial for the prevention of controlling the TORCH associated infection.

## Data Availability

The raw data supporting the conclusions of this article will be made available by the authors, without undue reservation.
